# Mechanism underlying the involvement of CXCR4/CXCL12 in diabetic wound healing and prospects for responsive hydrogel-loaded CXCR4 formulations

**DOI:** 10.3389/fphar.2025.1561112

**Published:** 2025-04-16

**Authors:** Lingli Wang, Fengsong Nie, Zhaoyu Lu, Yang Chong

**Affiliations:** ^1^ Department of Clinical Nutrition, The Affiliated Hospital of Yangzhou University, Yangzhou University, Jiangsu, China; ^2^ Institute of Translational Medicine, Medical College, Yangzhou University, Yangzhou, Jiangsu, China; ^3^ Department of General Surgery, The Affiliated Hospital of Yangzhou University, Yangzhou University, Jiangsu, China

**Keywords:** diabetic wound, CXCR4, CXCL12, mobilization, migration, agonist, AMD3100, responsive hydrogels

## Abstract

Diabetes mellitus is a prevalent chronic disease, often leading to complications, with chronic wounds being among the most challenging. Impairment of the CXCR4/CXCL12 signaling pathway, which plays a key role in cell mobilization, migration, and angiogenesis, significantly hampers the wound healing process in diabetic patients. Modulation of this pathway using CXCR4-targeted agents has shown promise in restoring wound repair capabilities. Additionally, the development of responsive hydrogels capable of adapting to external stimuli offers a powerful platform for drug delivery in chronic wound management. These hydrogels, when loaded with CXCR4 agonists or antagonists, enable controlled drug release and real-time therapeutic modulation. Integrating such hydrogels with existing wound healing strategies may provide an innovative and effective solution for overcoming the challenges associated with diabetic wound treatment.

## 1 Introduction

Diabetes mellitus, a chronic metabolic disease characterized by insufficient insulin secretion and/or insulin resistance (GBD, 2021 Diabetes Collaborators, 2023), is an intractable global public health problem. The concomitant increase in obesity and accelerated aging of populations, attributable to several factors, has led to a 102.9% increase in diabetes cases globally (Liu et al., 2020). The increase in the burden of disease has increased the economic burden. Currently, diabetes treatment is limited to glucose monitoring and pharmacological modulation, with a high risk of inducing systemic complications. The most prevalent complications include delayed or non-healing wounds, with a recurrence rate of up to 65% within 5 years, a 20% incidence of lower limb amputation, and a 10% mortality rate (Du et al., 2022; Armstrong et al., 2023; McDermott et al., 2023). The available treatment modalities for such wounds include debridement, anti-inflammatory agents, improvements in the oxygen supply, and flap grafting (Game, 2018; Wang M. et al., 2024). However, each of these methods has limitations in practical applications. These findings emphasize the need to optimize treatment strategies (Sun et al., 2024).

## 2 Wound healing process and characteristics of diabetic wounds

**FIGURE 1:**
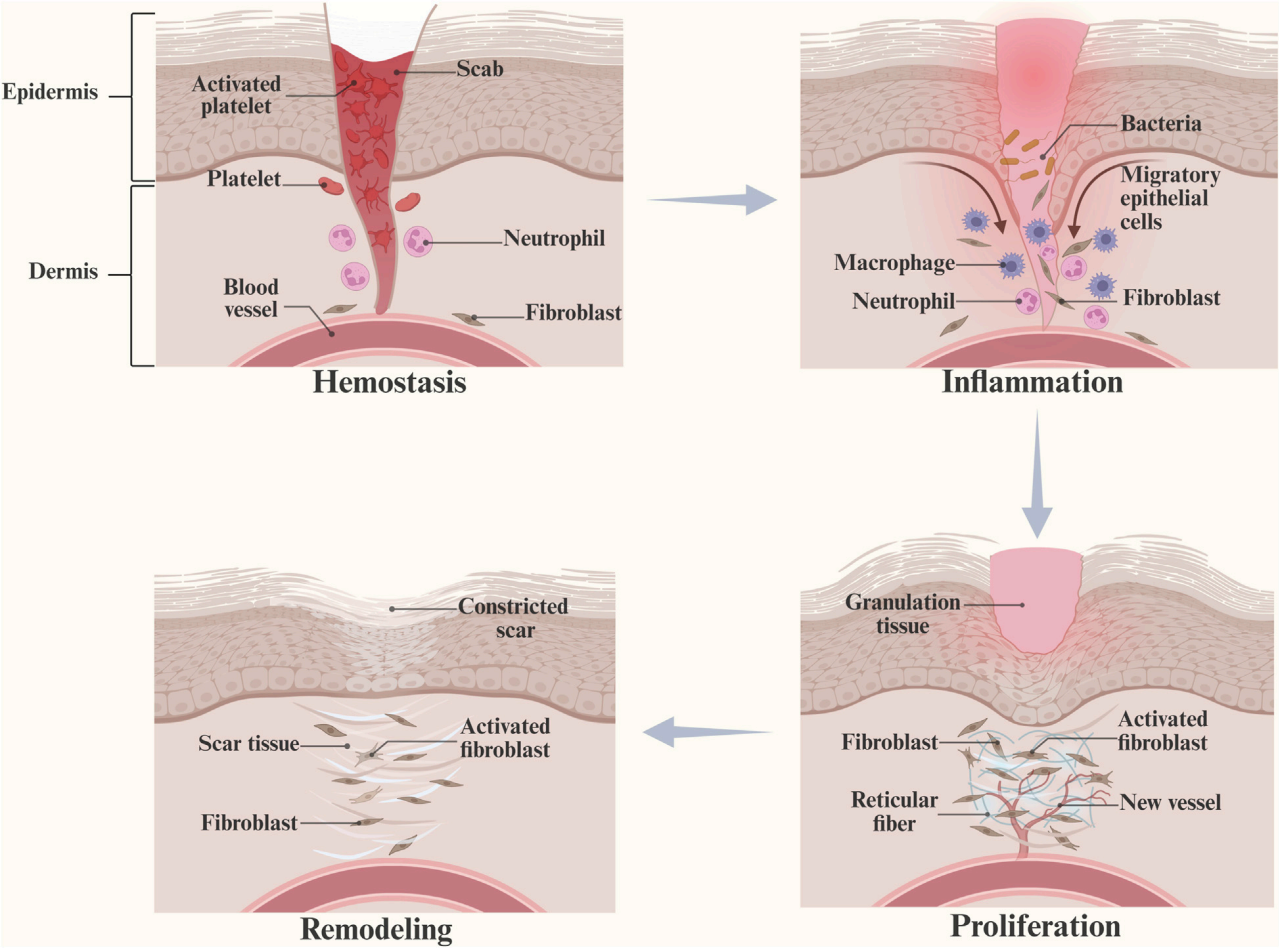
Hemostasis, inflammation, proliferation, and remodeling are four overlapping stages of wound healing. (This figure is created with BioRender.com).

**FIGURE 2:**
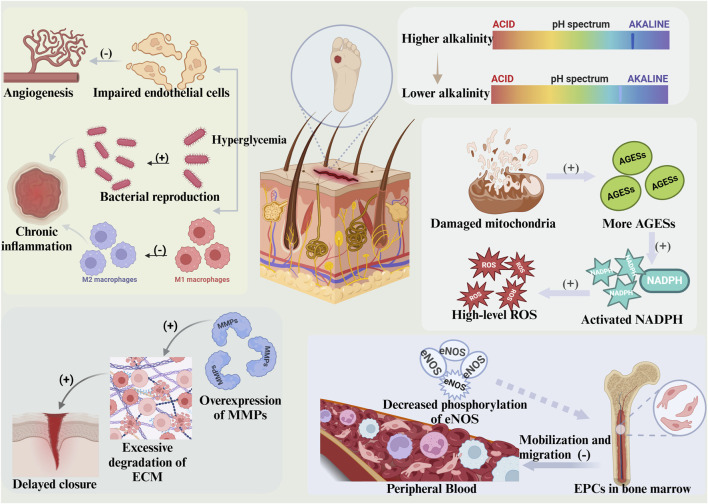
Diabetic wounds exhibit several characteristics. The pH of the wound remains alkaline for a prolonged period, with an initial pH that is elevated. Hyperglycemia can impede angiogenesis in the wound itself while concurrently promoting bacterial colonization and inhibiting macrophage polarisation, thereby triggering chronic inflammation. Impaired mitochondrial function produces excess AGEs, which activate NADPH and cause elevated levels of ROS. Overexpression of MMPs results in over-degradation of the ECM and subsequent delayed wound healing. The phosphorylation of eNOS is impaired, leading to decreased EPCs, affecting the mobilization of EPCs from the bone marrow and their migration to the peripheral blood. (This figure is created with BioRender.com).

Wound healing is a dynamic process that involves tissue regeneration and wound contraction through four overlapping phases, including hemostasis, inflammation, proliferation, and remodeling (Nosrati et al., 2023) (Figure 1). Following injury, platelets form fibrin clots at the site of injury (Pastar et al., 2014), which induce the synthesis of growth factors and promote the proliferation and migration of cells. Mast cells release serotonin and histamine to promote cell migration (Evans et al., 2013), and platelets release cytokines (PDGF, TGF-β, EGF, FGF, *etc.*) to promote local inflammation (Guo and Dipietro, 2010). Moreover, macrophages participate in phagocytosis and secrete cytokines and growth factors to stimulate granulation tissue formation (Li et al., 2007; Behm et al., 2012; Pakyari et al., 2013). In the proliferative phase, secreted factors increase vascular permeability and make the conditions suitable for recruiting endothelial, epidermal, and dermal cells to the peri-wound (Tottoli et al., 2020). During the angiogenic phase, collagen synthesis increases, granulation tissue is formed (Kaur et al., 2022), and IL-4 promotes the differentiation of fibroblasts into myofibroblasts (Fertin et al., 1991). When the remodeling phase starts with wound contraction, the induction of ECM production begins with the formation of granulation tissue and collagen degradation, followed by the production of new collagen and remodeling along the wound (Hinz, 2007). The newly formed vascular network rapidly develops into mature tissue structures, creating scar tissue (Cañedo-Dorantes and Cañedo-Ayala, 2019). Compared with normal wounds, diabetic wounds have the following characteristics (Figure 2).

### 2.1 Hyperglycemia

Hyperglycemia decreases blood flow to the wound, leading to endothelial cell damage and failure to initiate angiogenesis. It also provides favorable bacterial growth and proliferation conditions, exacerbating tissue hypoxia and inducing an inflammatory cascade. Switching macrophages from the pro-inflammatory M1 phenotype to the anti-inflammatory M2 phenotype is critical for wound healing. Excessive infiltration of M1 macrophages in diabetic wounds leads to the mobilization and migration of pro-inflammatory cells, triggering persistent inflammation. No FDA-approved drugs are known to target macrophages (Huelsboemer et al., 2024).

### 2.2 pH maintained at alkaline

The pH of the skin is weakly acidic on the surface and weakly alkaline inside. Regular wound healing shifts the pH from alkaline to acidic, but persistent inflammation in diabetic wounds contributes to a prolonged alkaline pH with a high initial pH.

### 2.3 Persistent hypoxia

Owing to the effects of inflammation, high glucose, prolonged hypoxia in wounds, and the inability of VEGF to induce eNOS phosphorylation and activate the CXCR4/CXCL12 signaling pathway, the weakened mobilization and migration of EPCs aggravate local inflammation and impair neovascularization. The oxygen supply to the ulcerated surface of the diabetic foot (DFU) is often improved clinically through hyperbaric oxygen therapy (HBOT), the delivery of proangiogenic factors, or the overexpression of stem cell factors.

### 2.4 High-level ROS

High glucose induces mitochondrial dysfunction, promotes advanced glycosylation end-product (AGE) formation, and activates NADPH oxidase, generating excessive reactive oxygen species (ROS) and disrupting the internal microenvironment. The infiltration of inflammatory cells damages cells and tissues, leading to impaired vascularization of the granulation tissue.

### 2.5 Overexpression of metalloproteinases (MMPs)

During wound healing, fibroblasts participate in the inflammatory response, degrading the damaged extracellular matrix (ECM), remodeling it by secreting MMPs, and promoting wound closure. In diabetic wounds, fibroblasts inhibit migration and increase the activity of secreted MMPs, leading to excessive degradation of the ECM and increasing the risk of chronic inflammation while compromising wound closure (Knoedler et al., 2023a).

## 3 Expression of CXCR4/CXCL12

### 3.1 Function of CXCR4 expression

The chemokine receptor CXCR4 is a highly conserved G protein-coupled receptor expressed in various cells, including stem cells, immune cells, epithelial cells, and cancer cells (Cano Sanchez et al., 2018; Fui et al., 2019). The gene sequence encoding CXCR4 is highly conserved between species, with 91% human-mouse homology. Knockout mice exhibit impaired functions, including impaired vascularization of organs (Ma et al., 1998; Tachibana et al., 1998; Singh et al., 2013). This finding suggests that the gene is indispensable in organisms (Giorgiutti et al., 2024). The expression and activity of CXCR4 are associated with tumor cell invasion, proliferation, migration, and the tumor microenvironment (Chen et al., 2024); blocking CXCR4 mobilizes leukemia cells from the bone marrow microenvironment (Xue et al., 2024). CXCR4 is also involved in the signaling required for HIV intervention and viral infection, promoting viral fusion (Zhou et al., 2024) and mediating the survival of hippocampal neurons in the central nervous system (Shan et al., 2021). Although the expression of CXCR4 may increase the risk of coronary heart disease and affect the degree of coronary artery stenosis (Li et al., 2023), it is involved in hematopoiesis, interventricular septum formation in terms of tissue regeneration, and the activation of stem cells (Huang et al., 2017), as well as neovascularization and the recovery of cardiac function after myocardial infarction (Ivins et al., 2015). The mobilization of stem cells, immune cells, *etc.*, and the migration of fibroblasts, endothelial cells, *etc.*, in which CXCR4 is involved, are closely related to wound healing (Wang F. et al., 2024).

### 3.2 Function of CXCL12 expression

CXCL12, also known as stromal cell-derived factor-1 (SDF-1) or myeloid pre-B-cell growth-stimulating factor (PBSF) (Rueda et al., 2012; Rossi et al., 2013), is widely expressed in the human lung, liver, kidney, and bone marrow (Cavallaro, 2013; Liepelt and Tacke, 2016). It is also involved in the proliferation and migration of tumor cells and is associated with chemoresistance (Meng et al., 2018). CXCL12 is involved in neovascularization, intimal hyperplasia, and thrombosis in atherosclerosis (Chai et al., 2022). Additionally, CXCL12 plays a role in neural stem cell migration, neuronal survival (Sowa et al., 2024)**,** and the pathogenesis of certain neurodegenerative diseases (Yan et al., 2022). Additionally, CXCL12 recruits immune cells to sites of inflammation, regulates inflammatory responses and cell migration (Zhong et al., 2024), and participates in wound healing (Wu et al., 2023).

### 3.3 CXCR4/CXCL12 signaling pathway

Although CXCR4 is not the only receptor for CXCL12, it is considered its primary receptor (Lechner et al., 2011). The binding of both triggers a conformational change in CXCR4, which promotes the activation of MAPK, PI3K/Akt, *etc.*, and calcium is released in a G protein-dependent manner through various signaling pathways (Blanchoin et al., 2014; D’Agostino et al., 2020), inducing a cellular response that maintains cell survival, mobilization, and migration (Scala, 2015; Giorgiutti et al., 2024). CXCR4/CXCL12 is widely expressed in various tissues and organs. It is involved in physiopathological processes such as growth and development, immunoinflammation, tumor invasion, wound healing, and fibrosis through various pathogenic mechanisms (Mimura-Yamamoto et al., 2017); CXCR4 is a promising therapeutic target (Wu et al., 2023). In chronic diabetic wounds, dysregulation of this signaling pathway is almost universally detrimental to healing. Thus, the modulation of this signaling pathway is expected to overcome the therapeutic dilemma of chronic wound healing.

## 4 Role of CXCR4/CXCL12 in diabetic wound healing

### 4.1 Involvement in mobilization

The injection of lentiviral vectors containing the CXCL12 inhibitory gene, which binds but does not activate CXCR4, exacerbates the early inflammatory response, and a reduction in the number of circulating EPCs inhibits neovascularization and the formation of granulation tissue (Bermudez et al., 2011). Induction of CXCR4 and CXCL12 expression in EPCs with cysteine-rich angiogenic inducer 61 (CCN1) can reverse hyperglycemia-induced impaired mobilization of EPCs (Dong et al., 2023). Similarly, injecting recombinant CXCL12 protein can amplify CXCL12 signaling in wounds and mobilize MSCs to overexpress CXCR4, which subsequently migrates to the wound surface to act (Hocking, 2015). Adipose tissue-derived stem cells (ADSCs) expressing CXCR4 were mobilized from wounds pretreated with CXCL12, and their number of survivors, wound closure index, and re-epithelial length improved (Li et al., 2016). Along with ADSCs, embryonic mesenchymal stem cell (EMSC)-produced CXCL12 also enhances wound healing by mobilizing CXCR4-expressing cells (Wang P. et al., 2019). Treatment with the CXCR4 antagonist AMD3100 increases the mobilization of progenitor cells (PCs) by more than six-fold (Allen et al., 2014). In addition to the above cells, neutrophils are also present. The TLR4 agonist monophosphoryl lipid A increases the mobilization of neutrophils. After severe burn injury, the expression of CXCR4 in bone marrow neutrophils decreases, whereas the plasma G-CSF concentration increases. This phenomenon can be explained by the fact that after induction by phospholipid A, G-CSF promotes neutrophil mobilization from the bone marrow to the bloodstream and the site of infection, generating an immune response (Bohannon et al., 2016). At the genetic level, placenta-specific gene 8 (Plac8) promotes the expression of CXCR4, which is a target of miR-181 and is involved in the mobilization and polarization of macrophages in ischemic tissues. In diabetic model mice, the deletion of miR-181 reduces the expression of CXCR4, decreasing the mobilization and polarization of macrophages to the M2 type, which is involved in tissue repair (Cheng et al., 2023). Impaired cell mobilization in diabetic wounds manifests through reduced mRNA and protein expression of CXCL12 (Peddibhotla et al., 2023). The construction of sustained delivery devices for CXCL12 in diabetic rat models, for example, the integration of such devices into scaffolds consisting of decellularized dermal matrix (DDM) and collagenous nanofibers, can effectively increase the number of CXCR4+ cells and increase their *in situ* mobilization (Amini et al., 2023). Pereira et al. (Pereira et al., 2024) suggested that the COAM-CXCL12 combination, consisting of a negatively charged straight-chain starch derivative, COAM, which binds to CXCL12 via electrostatic interactions, may increase the mobilization of EPCs by increasing their mobilization to and migration toward wounds, placing CXCL12 in the wound and preventing its rapid degradation by proteases. Moreover, tibial cortex transverse transport (TTT), a DFU surgical therapy developed by Ou et al. (Ou et al., 2023), increased the number of EPCs in the peripheral blood and the mRNA and protein expression of CXCR4 and CXCL12 at the level of skin ulceration. These findings indicate that the CXCR4/CXCL12 signaling pathway was successfully activated.

### 4.2 Participation in cell migration

A study (Xiao et al., 2020) reported that stem cell therapy and immunotherapy are promising avenues for treating chronic wounds. In addition to exerting direct therapeutic effects through stem cell differentiation, stem cell therapy can induce EPCs to migrate to the site of injury after mobilization from the bone marrow to the peripheral blood by increasing the secretion of vascular endothelial cytokines (e.g., CXCR4, MMP2/9, VEGF-α, and FGF2), thereby indirectly accelerating tissue repair. In addition to the previously mentioned ADSCs and MSCs, there are also bone marrow mesenchymal stem cells (BMSCs) (Huelsboemer et al., 2024) and regulatory T cells (Tregs) (2023; Knoedler et al., 2023b). At the same concentration gradient of CXCL12, CXCR4-overexpressing BMSCs had a significantly enhanced migratory capacity, whereas CXCR4-knockdown BMSCs had a reduced migratory capacity. The overexpression of CXCR4 is associated with insufficient expression of CXCL12 and prolonged wound healing (Yang et al., 2013). The migration of CXCR4+ cells to EMSCs was also inhibited after CXCL12 was knocked down in EMSCs (Wang X. et al., 2019). CXCR4/CXCL12 was found to promote the migration of EPCs to ischemic tissues in a mouse model of skin flap ischemia. In contrast, migrating EPCs secrete CXCL12, which mobilizes more EPCs to migrate to ischemic tissues (Tu et al., 2016). In terms of drugs, injecting untreated EPCs healed wounds in mice, whereas glucocorticoid (GC)-treated EPCs affected wound healing via the mechanism by which GC inhibited the migration of EPCs to CXCL12 by downregulating CXCR4 (Carolina et al., 2018). A study (Lin et al., 2021) suggested that granulocyte colony-stimulating factor (G-CSF) may promote the migration of CXCL12+ cells from the bone marrow to the peripheral blood via the CXCR4/CXCL12 signaling pathway to promote subsequent angiogenesis, as convinced by a significant increase in CXCL12 in the wounds of diabetic mice in the G-CSF alone group. Additionally, culturing EPCs on MCSs (Deshpande et al., 2018) or local transplantation of EPCs after autologous isolation (Leng et al., 2021) can stimulate the functional restoration of the CXCR4/CXCL12 signaling pathway during the migration of EPCs, which in turn accelerates wound healing. In chronic diabetic wounds, Tregs have advantages and disadvantages; inhibiting their migration leads to excessive inflammatory responses and delayed wound healing. Depletion of Tregs, in turn, prevents hyperfibrosis and scarring by inhibiting TGF-β activity, contributing to the remodeling process (Knoedler et al., 2023b). In type I diabetic (T1DM) mouse wounds, the expression levels of the CXCR4/CXCL12 and VEGF mRNAs and proteins and the wound healing rate are significantly lower than those in normal wounds (Jing et al., 2015). Despite low levels of expression of CXCL12, the migration of Gr-1+CD11b+ myeloid cells to the wound was not affected, although migration was inhibited after the administration of AMD3100 (Tong et al., 2014). A previous study (Allen et al., 2014) reported that AMD3100 inhibits PC migration and affects angiogenesis and wound healing. In addition to stem cells and PCs, epithelial cells are present. *Morus alba* root extract (MA) treatment can promote keratinocyte proliferation, migration, and epithelial cell growth by increasing the levels of CXCR4 and CXCL12 (Kim et al., 2015). CXCR4/CXCL12 also exerts a positive effect (Yellowley et al., 2019). Treatment with antimicrobial peptides (AMPs) promotes the proliferation and migration of fibroblasts and induces the expression of genes associated with tissue repair. Two AMPs (clavanin-A and mastoparan-MO) can induce wound closure and accelerate wound healing (Alencar-Silva et al., 2024).

### 4.3 Involvement in neovascularization

Angiogenesis occurs mainly in the inflammatory and proliferative phases. Following normal skin wound formation, HIF-1α is upregulated through hypoxia and the PI3K/Akt signaling pathway, leading to the upregulation of downstream angiogenesis-related factors and the promotion of angiogenesis (Jing et al., 2015). Increased expression of CXCL12 in wounds is accompanied by increased release of endogenous bone marrow progenitor cells (BM-PCs) induced by the CXCR4/CXCL12 signaling pathway and accelerated angiogenesis (Fiorina et al., 2010). Decreased endothelial cell proliferation and an attenuated response between cells and growth factors lead to insufficient angiogenesis in diabetic wounds (Tahergorabi and Khazaei, 2012). CXCL12 is inhibited in hyperglycemia, neointima formation is blocked (Grunewald et al., 2006), and M2-type macrophages cannot produce cytokines that promote angiogenesis. Consequently, neovascularisation in diabetic wounds is characterized by slow and limited proliferation of new blood vessels. (Cheng et al., 2023). The protein p21Cip1 is an essential mediator of CXCL12 transcription, and inhibition of CXCL12 significantly reduces neointima formation after vascular injury in p21−/− mice. Neointima formation in p21+/+ mice is also considerably inhibited when the expression of CXCR4 is blocked (Olive et al., 2008). These findings suggest that CXCR4/CXCL12 constitute a signaling pathway. Relaxin (RLX) significantly shortens the time needed to close diabetic wounds. However, if antibodies against CXCR4 are administered simultaneously, they affect angiogenesis and decrease the efficacy of RLX (Bitto et al., 2013). Additionally, treatment with simvastatin or BMSCs improves healing, and the combination of the two has a synergistic effect, increasing the expression of CXCL12 and CXCR4, which promote the differentiation of epithelial and endothelial cells for epithelial reformation and angiogenesis, respectively (Mohajer Ansari et al., 2020). Partial CXCR4 agonist treatment significantly increased the number of neovascularizations in a diabetic mouse wound model. For example, topical application of UCUF-728 activates CXCR4 to promote angiogenesis and shorten the time to wound closure by inhibiting miR-15 b (Xu J. et al., 2022). At the genetic level, amniotic membrane mesenchymal stem cells (hAMSCs) modified by double knock-in of the angiogenic/anti-inflammatory genes CXCR4/IL10 significantly increase the tube length and branching number of wound tissues, and the angiogenic capacity increases (Han et al., 2022). CD31, a marker of vascular endothelial cells, is closely associated with angiogenesis (Dai et al., 2024). Nanomaterials loaded with CXCL12 significantly increased the density of CXCR4+ cells during the first 21 days of wound healing. Highly vascularized and organized granulation tissue was observed on day 7, with a substantial increase in the number of *in situ* blood vessels and a reduction in the area covered by the wound (Amini et al., 2023; Peddibhotla et al., 2023). Qin et al. investigated the study conducted by Ou et al. (Ou et al., 2023). They reported that TTT increased the expression of proangiogenic factors with significantly increased serum levels, which is essential for neovascularization. They suggested that the mechanism underlying this promotion is attributed to the activation of the CXCR4/CXCL12 signaling pathway (Qin et al., 2024).

## 5 CXCR4 formulation for diabetic wound healing

### 5.1 CXCR4 antagonists

Among CXCR4 antagonists (Table 1), only AMD3100 (plerixafor) is approved by the FDA for clinical use. AMD3100 significantly reduces leukocyte mobilization, consequently leading to scarless repair through the suppression of inflammation and the promotion of angiogenesis. (Leung et al., 2015). AMD3100 also promotes wound healing with G-CSF. The former promotes the mobilization of EPCs in the bone marrow, and the latter promotes their migration. Additionally, AMD3100 (Lin et al., 2021) may also be associated with an increase in the mobilization of inflammatory cells on day 3. Similar to GC, AMD3100 disrupts the CXCR4/CXCL12 signaling pathway, inhibits epidermal stem cell migration (Guo et al., 2015), prevents the migration of BMSCs and EPCs (Hu et al., 2013), and delays wound healing (Carolina et al., 2018). As proposed by Allen et al. (Allen et al., 2014), while AMD3100 treatment resulted in a more than six-fold increase in PC mobilization, the migratory capacity decreased by 25.1%. Moreover, despite an increase in the mobilization of PCs by AMD3100, treatment alone only partially restored (70%) neovascularization in diabetic wounds, and this impaired migration was ameliorated in combination with PDGF-BB. These findings suggest that AMD3100 treatment may impair the function of PCs or that an increase in the mobilization of PCs alone cannot overcome defects in diabetic neovascularization. In a phase IIa clinical study, the researchers enrolled 26 patients and used complete healing at 6 months as the primary outcome measure. The study was prematurely terminated after 3 months because of a significantly lower healing rate in the plerixafor group. The results revealed a healing rate of 69.2% in the placebo (saline) group and only 38.5% in the plerixafor group, despite the successful mobilization of hematopoietic stem and progenitor cells (HSPCs) in the plerixafor group. The secondary outcome indicators of wound size, transcutaneous oxygen tension (TcO2), the ankle-brachial index (ABI), and the amputation rate were not significantly different, and incidental adverse events (rash, acute coronary syndrome) were not associated with plerixafor (Bonora et al., 2020). In another phase I clinical trial, MRG-001, which consists of plerixafor and tacrolimus, mobilized a wide range of stem and immunomodulatory cells into the peripheral blood at doses ranging from 0.005 to 0.2 mL/kg, with the most favorable results at 0.01 mL/kg. Neither drug reached the toxic or immunosuppression thresholds (Ahmadi et al., 2023). In summary, AMD3100 exerts dual effects on diabetic wound healing, probably by promoting wound healing. It may promote wound healing by facilitating stem/progenitor cell mobilization, modulating inflammatory responses, inhibiting hyperfibrosis, and potentially achieving scarless healing. It also affects neoangiogenesis by inhibiting cell migration, which hurts wound healing. Additionally, AMD3100 has been used in antitumor studies, as CXCR4/CXCL12 is highly expressed in specific tumor cells. Owing to its dual effects on wound healing, researchers have raised concerns regarding its safety. In patients with tumors in combination with chronic non-healing trauma or with DFUs that contain tumors, the conditions of application are more stringent. In such cases, strict adherence to dosing guidelines is needed, or an alternative therapeutic approach must be selected.

**TABLE 1:** CXCR4 agonists and antagonists in diabetic wound healing.

Category	Name	Advantages	Disadvantages	Application phase	References
CXCR4 agonist	UCUF-728	Promote fibroblast migration and reduce the expression of genes and proteins that inhibit angiogenesis and collagen production.	There is a short observation period and no clinical studies.	Animal	Xu et al. (2022b)
	UCUF-965	The maximal effect of CXCL12 was enhanced by 208% at 10uM administration, promoting neovascularisation and wound closure without side effects.	The optimal dose safety range and risk of increased fibrosis and scarring are not determined.	Animal	Peddibhotla et al. (2023)
CXCR4 antagonist	AMD3100	6-fold increase in mobilization of PCs from bone marrow to peripheral blood.	25.1% reduction in migratory capacity and mobilization of PCs alone cannot meet healing requirements.	Animal	Allen et al. (2014)
		Reduces leukocyte mobilization, inhibits the inflammatory response, and promotes angiogenesis for scarless healing.	There are no clinical trials.	Animal	Leung et al. (2015)
			It inhibits the migration of epidermal stem cells and affects wound healing.	Animal	Guo et al. (2015)
		Promoting mobilization of inflammatory cells in the early stages of wound healing.		Animal	Lin et al. (2021)
			The experiment was terminated early due to the small sample size and heterogeneity, and the drug interfered with wound healing.	Phase IIa Clinical	Bonora et al. (2020)
		Mobilizing stem and immune cells into the peripheral bloodstream with tacrolimus achieves dual pro-healing and non-toxicity.	Lack of clarity on optimal dosage and dosing regimen and ambiguity on the mechanism of combined effects.	Phase I Clinical	Ahmadi et al. (2023)

### 5.2 CXCR4 agonists

CXCR4 agonists are still in the animal research stage. *In vitro* studies conducted by Xu et al. (Xu Y. et al., 2022) indicated that, unlike AMD3100, which replaces CXCL12, UCUF-728 enhances CXCL12 signaling by decreasing the expression of microRNAs (miRs) 15b and 29a. These microRNAs inhibit angiogenesis and collagen production in diabetic fibroblasts. Additionally, UCUF-728 partially activates CXCR4 and promotes wound healing by stimulating cell migration. In a diabetic mouse wound model, the effects of UCUF-728 were observed for 22 days, and the results revealed an increase in neovascularization by day 7. Peddibhotla et al. (2023) reported a 208% increase in the maximal effect of CXCL12 when the CXCR4 agonist UCUF-965 was administered at a concentration of 10 μM, without significant toxicity or adverse effects. Histological staining revealed increased neovascularization, and the wounds healed completely within 14 days. Both methods decreased the time to wound closure by 36%. However, UCUF-965 was 10 times more potent than UCUF-728 in activating CXCR4-mediated cell mobilization, migration, and maximal effects of CXCL12; it also showed more significant therapeutic potential in promoting wound healing.

## 6 Hydrogel dressings for the healing of multiple wounds

With advancements in research, the choice of wound dressing has diversified along with the modulation of the CXCR4/CXCL12 signaling pathway to improve diabetic wound healing. Traditional dry dressings, such as gauze, have limitations, including causing wound dehydration, impeding cell migration, and reducing the efficiency of antimicrobial agents. They often adhere to neoplastic granulation tissue and cause secondary injury during replacement. Ideal wound dressings should have features that provide a microenvironment that favors wound healing while effectively absorbing wound exudate (Tavakoli and Klar, 2020). On the basis of the theory of moist environment healing, researchers have developed wound dressings such as films, foams, and hydrogels (Hilton et al., 2004; Shi et al., 2020; Spampinato et al., 2020)**.** Among them, hydrogel dressings have good adhesion ability, excellent hydrophilicity, good drug-carrying capacity, and a unique three-dimensional porous structure (Chen et al., 2023). In addition to their role in managing chronic wounds related to diabetes, hydrogels are crucial for treating trauma and thermal burns. Cross-linking hydrogel (CNG) is a light-responsive hydrogel used as a barrier material to prevent postoperative adhesions and reduce the incidence of tissue adhesions (Yang et al., 2017). In the context of traumatic brain injury (TBI), hydrogels are used primarily as carriers for stem cells. For example, the inoculation of human umbilical cord mesenchymal stem cells (hUC-MSCs) that express CXCR4, as well as astrocytes combined with RADA16-BDNF (Shi et al., 2016) and an SA/Col/CXCL12 hydrogel loaded with BMSCs (Ma et al., 2021), was found to facilitate stem cell migration. This process requires the CXCR4/CXCL12 signaling pathway and its downstream effectors, including FAK, PI3K, and AKT, which are necessary for neural repair following TBI. In the thermal burn module, the three-dimensional network structure of the hydrogel contributes to its high water absorbency, effectively preventing wound adhesion due to dryness (Goh et al., 2024). Its appropriate mechanical strength helps the wound resist external mechanical stimuli (Yao et al., 2025). A previous study (Shu et al., 2021) revealed that while skin substitutes are created *in vitro*, better deposition of the ECM in the scaffolds can be achieved by gradually degrading the hydrogel surrounding the scaffold. This process promotes the healing of burn wounds and prevents scar formation. With respect to the role of the signaling pathway in the healing of burn wounds, the use of hydrogels to modulate CXCR4/CXCL12 has not been addressed in studies that are predominantly limited to pharmacological modulation. Following burn injury, monocytes are recruited to the wound, promoting fibroblast activation and eventual hypertrophic scarring (HTS), which is attenuated by the CXCR4 antagonist CTCE-9908 (Ding et al., 2011).

In addition to traumatic and thermal burns, the spectrum of burn injuries also includes chemical burns. CXCR4 expression increases considerably and is normalized by tetramethylpyrazine (TMP) treatment in a mouse corneal alkali burn model. This phenomenon may be attributed to the inhibitory effect of TMP on pathological CXCR4, which is achieved by suppressing the expression of NRF-1 during corneal neovascularization (CNV) (Tang et al., 2018). Another study revealed that a carbomer-based ferulic acid (FA) hydrogel significantly ameliorated radiation-induced skin damage by inhibiting the activation of the NLRP3 inflammasome and reducing oxidative stress (Huang et al., 2024).

## 7 Responsive hydrogels for diabetic wound application and adverse effects

**FIGURE 3:**
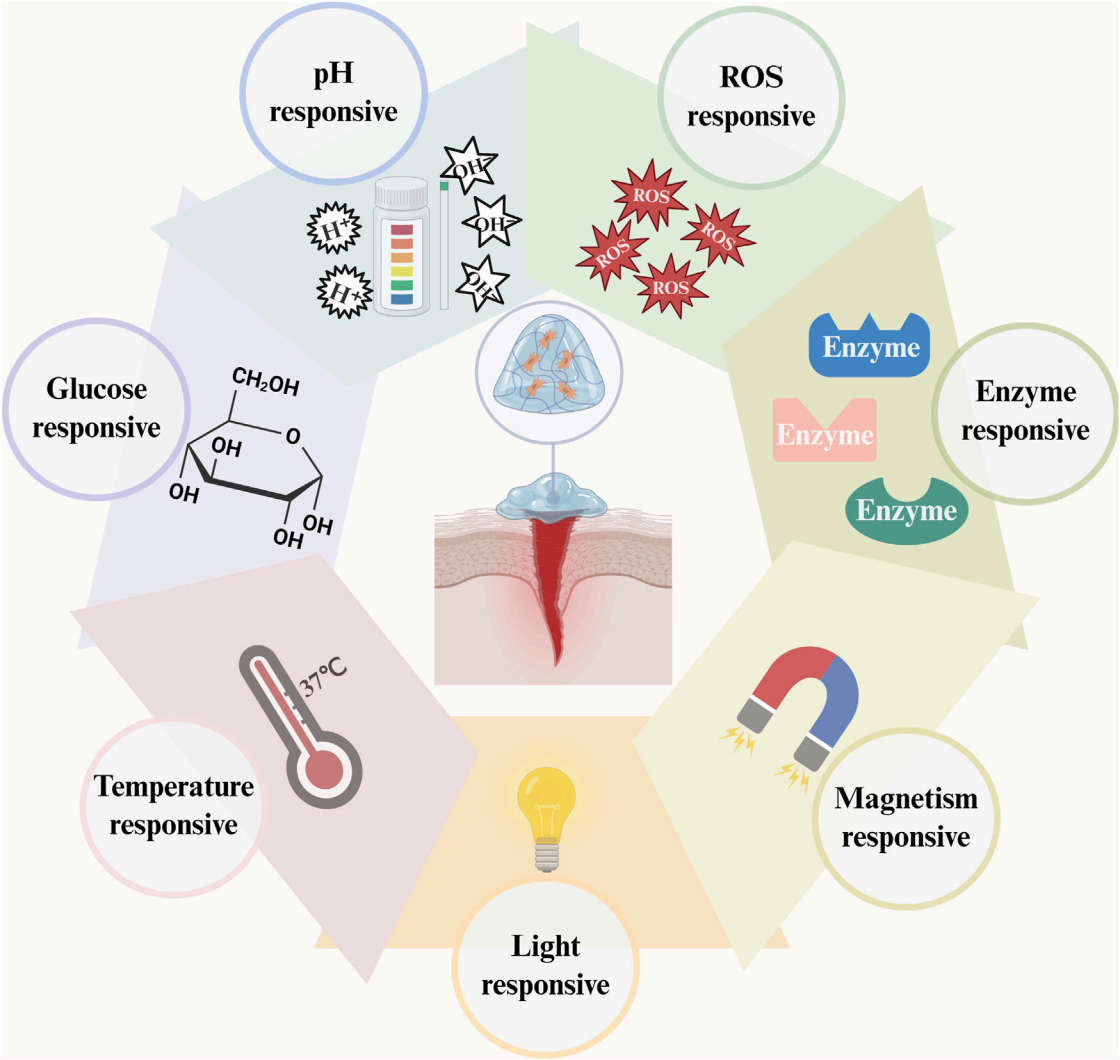
The primary determinants of hydrogel responsiveness encompass pH, ROS, enzymes, magnetism, light, temperature, and glucose concentrations. (This figure is created with BioRender.com).

Conventional hydrogels combine external stimuli and bioactive components to form responsive hydrogels (Figure 3) that achieve targeted drug release and controlled degradation by responding to single or multiple factors, such as glucose, pH, ROS, enzymes, light, magnetism, and temperature, to regulate wound healing precisely (Chen et al., 2023) (Table 2). The glucose-responsive QL@MAB developed by Liu et al. (Liu et al., 2025) functionally and structurally mimics human skin, precisely releases drugs by breaking borate bonds, rapidly eliminates bacteria, and promotes angiogenesis. The pH/ROS/glucose-responsive hydrogel CPO/D@P/IGF-1C developed by Dai et al. (Dai et al., 2024) is also photothermally stable and promotes wound healing through multiple mechanisms. LZMMPHEP has MMP-2-responsive growth factor release properties with more efficient VEGF release (Huang et al., 2020). In contrast, GelMA loaded with t-ZnO/VEGF achieves photoresponsive growth factor release and promotes angiogenesis (Siebert et al., 2021). In diabetic mice, wireless magneto-induced dynamic mechanical stimulation (MDMS) combined with cell therapy and a magnetoresponsive hydrogel to form a therapeutic platform significantly accelerated wound closure (92.7%) (Shou et al., 2023). The photoresponsive/thermoresponsive hydrogel developed by Wang et al. can achieve controlled drug release (Wang L. et al., 2024). BPCH-B showed remarkable drug release ability at pH 7.6°C and 40°C, with a greater than 70% release rate and good biodegradability (Zhang et al., 2023). Most hydrogels have poor mechanical properties. However, the self-growing hydrogel bioadhesive (sGHB) developed by Zheng et al. (Zheng et al., 2024) has good mechanical properties; sGHB not only mimics the self-growth behavior of biological tissues but also reduces the wound dilatation rate by up to 30%, enabling dynamic mechanical regulation of the wound. The pH-responsive hyaluronic acid hydrogel has good stability and mechanical strength. It can quickly (50 s) rebuild and restore the original properties after fracture, extending the service life (Zhang et al., 2023). D-MAP hydrogels induce tissue repair by activating adaptive immunity and healing wounds with increased tensile strength (Griffin et al., 2021). The cumulative acid (AA)-loaded hydrogel exhibited improved swelling properties and thermal stability, accelerating the immune response and re-epithelialization in a zebrafish model (Kandaswamy et al., 2025). M@M-Ag-Sil-MA for diabetic orthopedic surgical wounds promotes phenotypic shifts in macrophages, migration of fibroblasts, and neovascularization for spatiotemporal immunomodulation (Mei et al., 2022).

**TABLE 2:** Multiple responsive hydrogels for diabetic wound healing studies.

Name	Components	Response factor	Characteristics	References
LZ-MMPHEP	Leucine Zipper, ESQES and QESQSEQDS, LRKKLGKA, IPVSLRSG	Enzyme	It has improved mechanical properties and biocompatibility. The slow release of growth factors promotes the proliferation and differentiation of HMSCs and accelerates vascularisation.	Huang et al. (2020)
MAP	Microgel Building, L-MAP or D-MAP	Enzyme	D-MAP recruits immune cells and triggers adaptive immunity. It has a fast degradation rate in the body with no residue after wound healing. It also increases skin thickness, tensile strength, and neoplastic hair follicles and sebaceous glands.	Griffin et al. (2021)
M@M–Ag–Sil-MA	Silk Fibroin, Methacryloyl groups, Photoinitiator (LPA), MET@ MSNs, Ag NPs	Light, pH	It has good dispersion, homogeneity, mechanical stability, and biocompatibility. The strong antimicrobial capacity helps promote macrophage polarisation for immunomodulation and accelerates angiogenesis.	Mei et al. (2022)
Dual-crosslinked Hyaluronic Acid Hydrogel	AMHA, DTP, Darocur 2959 (D-2959)	Light	It gels rapidly, increasing mechanical strength with no cytotoxic, but has not been tested *in vivo*.	Zhang et al. (2023)
CPO/D@P/IGF-1C	CMCS-PBA, OXD, DFO@PDANP(D@P), IGF-1C	pH/ROS/Glucose, Temperature	It is injectable, photothermally stable, temperature-responsive, has good antimicrobial activity, and regulates blood glucose levels through the slow release of drugs, anti-inflammatory, and antioxidants.	Dai et al. (2024)
Self-growing Hydrogel Bioadhesive (sGHB)	PAHN, Gelatin, PEGDA, Gox, FeCl_2_·4H_2_O, Deionized water	Glucose	It has good bioadhesive properties and effectively inhibits inflammatory reactions, promoting wound collagen deposition and reepithelization and avoiding wound expansion.	Zheng et al. (2024)

Specific components of hydrogels, including natural polymers, synthetic polymers, cross-linking agents, plasticizers, and drug carriers, may pose risks or elicit adverse reactions. Four studies involved hydrogels containing alginate (Punnoy et al., 2024), polyacrylic acid (Wang et al., 2023), glutaraldehyde (Pratinthong et al., 2024), oleic acid (Gallelli et al., 2020), and PLGA (Bastidas et al., 2023) as different components. The findings of these studies indicated that the hydrogels exhibited favorable biocompatibility in animal models and did not elicit adverse reactions, such as allergies, pain, or scarring. Additionally, pH-responsive ZIF-90@i-PPOPs (Li et al., 2024), SAB (Song et al., 2025), and temperature-responsive CPAG (Cai et al., 2023) showed no signs of secondary infections in animal models. The temperature-sensitive hydrogels developed by Dadkha et al. (Dadkhah Tehrani et al., 2022) and the thermoresponsive hydrogels showed no hemolytic reactions or indications of toxicity. In clinical studies, Humbert et al. (Humbert et al., 2014) reported that HydroClean^®^ significantly reduced the necrotic tissue content while increasing the granulation tissue content in venous leg ulcer wounds. Additionally, seven out of 15 patients treated with HydroClean^®^ reported experiencing pain, possibly due to age, wound complexity, and skin sensitivity. A subsequent systematic review that included 272 patients revealed that although no adverse reactions or side effects were reported, it is still premature to consider hydrogel dressings more effectively than alternative treatments for venous leg ulcers. This conclusion was based on limited sample size, subpar quality of evidence, and a lack of long-term follow-up data (Ribeiro et al., 2022). This study revealed that during long-term use, regardless of whether the cellular components in the hydrogel were of autologous (Nilforoushzadeh et al., 2020) or allogeneic (Moon et al., 2019) origin, there were no secondary adverse reactions, such as inflammation necrosis or immune rejection. Clinical studies in which hydrogels promote wound healing remain limited, with even fewer studies on long-term follow-up. However, these studies’ absence of adverse reactions and side effects suggests that immunogenicity and secondary inflammation have not been demonstrated over longer treatment cycles. Many antitumor studies have used ROS-responsive hydrogels. For example, ROS can improve the immunogenicity of PIVOT, triggering innate and adaptive immune responses and achieving a tumor inhibition rate of up to 90% (Fu et al., 2024). Therefore, this hydrogel may reduce the chances of recurrence and metastasis by enhancing the tumor microenvironment, boosting the immune system, facilitating targeted drug delivery, and enabling *in situ* injection after tumor resection. These findings indicate the significant clinical potential of ROS-responsive hydrogels.

## 8 Hydrogels in conjunction with other diabetic wound-healing strategies

### 8.1 Negative pressure wound therapy (NPWT)

Negative pressure wound therapy (NPWT) is an effective strategy for promoting wound healing. However, if the foam pad is covered for an extended period and the wound is left unobserved, NPWT can increase the risk of infection and sepsis. Additionally, NPWT may destroy neoplastic granulation tissue when it replaces padding. Conversely, injectable porous hydrogel scaffolds may be a suitable alternative, although their structural stability and mechanical properties are inferior (Staruch et al., 2017). In a previous study (Rawson et al., 2023), a novel NPWT foam material (V.A.C.^®^ Granufoam™) loaded with the antimicrobial agent CZ-01179 showed a strong antimicrobial effect against methicillin-resistant *Staphylococcus aureus* (MRSA) and *Acinetobacter baumannii*. The hydrogel scaffold not only functions as a carrier for antimicrobial agents but also mimics the function of the natural ECM, thereby facilitating the adhesion, proliferation, and differentiation of cells.

### 8.2 Hyperbaric oxygen therapy (HBOT)

Chronic diabetic wounds are characterized by ongoing hypoxia, which is why HBOT is provided in clinical settings; however, it is expensive. *In vitro* studies have shown that a hybrid oxygen-producing wound dressing using a decellularized amniotic membrane (dAM) combined with a temperature-sensitive hydrogel made of chitosan promotes cell growth and adhesion. It also has a low incidence of short-term adverse effects, such as hemolysis, indicating strong potential for clinical application (Dadkhah Tehrani et al., 2022). The results of the animal experiments revealed that three oxygenated hydrogels, P407, P407/P188, and nanocellulose-based gel (NCG), with high levels of dissolved oxygen effectively reduced inflammation during wound healing from days 3–10. Additionally, they significantly promoted re-epithelialization as early as day 3 (Tan et al., 2024). This local oxygen delivery system can address the drawbacks of expensive HBOT while promoting healing. However, concerns regarding the stability and continuity of oxygen release, uniform dispersion of particles, and potential long-term toxicity need to be addressed. No clinical trials have been conducted to validate these issues, and the feasibility of clinical application remains uncertain.

### 8.3 Electrical stimulation therapy (EST)

Electrical stimulation therapy (EST) accelerates wound healing by promoting cell proliferation and migration. Phosphatase and tensin homolog (PTEN) is a protein commonly found in somatic cells that influences cellular electrical responses; however, it also inhibits cell proliferation, migration, and differentiation. Therefore, inhibiting PTEN can improve the efficacy of EST in wound healing. An electroresponsive hydrogel loaded with a PTEN inhibitor (BPV@PCP), as investigated in a previous study (Fu et al., 2023), was able to attach to a wearable direct pulse piezoelectric nanogenerator (PENG). This device converts motion into an electric field, which triggers the on-demand release of PTEN inhibitors. Researchers reported a 21.33% increase in the rate of whole-layer skin healing in the rats investigated. This effect was mediated by the activation of the PI3K-Akt pathway, a downstream signal of CXCR4/CXCL12. Another study (Qi et al., 2024) examined the effectiveness of three geometries of flexible microelectrodes (FMs), including hexagonal, cross-shaped, and serpentine, in promoting electrical stimulation (ES) for chronic wound healing. In animal experiments, researchers have integrated these three types of FMs with hydrogel dressings made from PPY, PDA, and PANI. The findings indicated that the serpentine geometry provided the most incredible flexibility, safety, and healing efficiency, achieving a wound closure rate of up to 91% by day 10.

### 8.4 Phototherapy

Phototherapy is a promising strategy for managing microbial infections and includes techniques such as photodynamic therapy (PDT) and photothermal therapy (PTT). PDT uses low-intensity visible light (VL) or near-infrared (NIR) light to activate photosensitizers (PSs). This activation leads to the generation of ROS, which disrupts bacterial cell membranes. Natural reticulated hydrogels, including chitosan (Cs), collagen, alginic acid, and hyaluronic acid, are effective in PDT because of their excellent degradability, biocompatibility, and low toxicity (Xu Y. et al., 2022). Curcumin (Cur) is a naturally occurring PS that enhances the properties of the thermosensitive hydrogel TQ@PEG-PAF-Cur. This combination helps combat multidrug-resistant bacterial infections and supports the reconstruction of the surrounding microenvironment (Zhao et al., 2024). In contrast, synthetic reticulated hydrogels made from polyvinyl alcohol (PVA), polymethacrylic acid, and polyacrylamide show superior water absorption, greater mechanical strength, and longer service life (Zhao et al., 2024). The PTT mechanism involves hydrogel light absorption and then the conversion of the absorbed light into heat. The heat generated disrupts bacterial cell membranes by increasing the local temperature. The synergistic effect of using Cur and PTT can enhance wound healing and increase antimicrobial activity, mainly because the photothermal effect facilitates the transfer and release of photosensitizers. However, further optimization is needed for ROS generation by photosensitizers and increasing the photothermal conversion efficiency of photothermal agents (Xu Y. et al., 2022). A research group (Sun et al., 2023) developed a novel photonic hydrogel by incorporating nanohydrogels into a dual-network structure. This hydrogel uses the combined effects of NIR light-induced thermal energy and ROS to achieve photosensitivity and photothermal effects, enabling rapid sterilization at temperatures below 50°C. Following the findings of a recent research report, another research group (Yao et al., 2025) designed silk fibroin (SF) hydrogels based on gold (Au) nanoparticles modified with metal-organic frameworks (MOFs). These hydrogels combined dual PTT and PDT therapeutic techniques, resulting in a uniform distribution of nanoparticles and improved photothermal conversion efficiency, ROS generation, and wound healing rates of up to 99.06%.

### 8.5 Cells and growth factors

Hydrogels are used as carriers for cells and growth factors in wound healing. Notable examples include hPL (Lim et al., 2020), EGF (Pessanha et al., 2023), NGF and VEGF (Li et al., 2021), PDGF-BB (Banerjee et al., 2021), and ADSCs (Min et al., 2025). However, the molecular mechanisms underlying tissue regeneration induced by hydrogel-delivered growth factors must be further elucidated. The stability of growth factors placed in hydrogels also needs improvement, and more precise measurements of the release kinetics of these growth factors are necessary to accommodate various clinical needs (Shan and Wu, 2024).

## 9 Limitations and prospects

Studies have shown that the inhibition of CXCL12 expression due to elevated glucose levels results in the dysfunction of CXCR4 and a decrease in the number of circulating EPCs following impaired mobilization of EPCs. Administering CCN1 can promote the expression of CXCR4 and CXCL12 in EPCs and reverse impaired mobilization of EPCs. The supplementation of CXCL12 and the amplification of its signals can mobilize MSCs, ADSCs, and EMs and promote their expression of CXCR4, thereby facilitating wound healing. These findings suggest that stem cells can act as a bridge between CXCR4 and CXCL12. The CXCR4 antagonist AMD3100 facilitates the mobilization of PCs from the bone marrow to the peripheral blood, thereby positively influencing wound healing. Additionally, deleting miR-181 decreases the expression of CXCR4, and impaired CXCR4/CXCL12 affects wound healing by promoting inflammatory responses. At the molecular level, impaired CXCR4/CXCL12 expression is associated with a reduction in the CXCR4/CXCL12 mRNA and protein levels. Ou et al. (Ou et al., 2023) reported that surgical TTT promotes the expression of both proteins and facilitates wound healing by increasing the number of EPCs in the peripheral blood. However, preliminary evidence suggests that TTT can effectively treat DFU; the experiments conducted to obtain relevant results have several limitations. First, it had a short observation period (21 days). Second, the sample size was small (18 rats per group). Third, the molecular mechanism by which the CXCR4/CXCL12 signaling pathway regulates the accumulation of EPCs and promotes wound healing has not been elucidated. Finally, the study did not validate the relationship between surgery and the use of agonists or antagonists of CXCL12 or CXCR4 through the causality of wound healing. Additionally, long-term wound stability, recurrence rates, chronic complications (pain, bleeding, edema, and infection), and functional recovery of the wound were not considered.

Impaired cell migration in diabetic wounds is characterized primarily by reduced mRNA and protein expression levels of CXCR4 and CXCL12. The downregulation of CXCR4 by GCs affects the expression of CXCL12, and conversely, knocking down CXCL12 in EMSCs can influence the function of CXCR4. At a constant concentration of CXCR4, high or low levels of CXCR4 expression differentially affect the migration of BMSCs and EPCs. These findings indicate that CXCR4/CXCL12 is necessary for regulating the migration of stem cells. Along with stem cells, the migration of epidermal cells and fibroblasts, promoted by CXCR4/CXCL12, plays an active role in wound healing. AMPs induce the expression of genes associated with tissue repair by promoting the migration of fibroblasts. Additionally, inhibiting Treg migration significantly delays wound healing in diabetic patients. Supplementing EPCs via exogenous routes, such as matrix embedding and local transplantation after autologous isolation, can stimulate the functional recovery of CXCR4/CXCL12 in diabetic wounds. Tu et al. (2016) reported positive feedback regulation between CXCR4, CXCL12, and EPCs, with the first two constituting a signaling pathway that promotes the migration of EPCs, and migrating EPCs, in turn, can mobilize CXCR4+ cells for additional migration.

Persistent hyperglycemia inhibits CXCL12 expression in diabetic wounds. This condition can also cause macrophages to remain in the pro-inflammatory M1 type, impair their polarization, and render M2-type macrophages unable to produce proangiogenic factors. These mechanisms of action affect wound healing. Administering exogenous drugs such as RLX and simvastatin improved vascularization and wound closure. Additionally, the knock-in of anti-inflammatory/angiogenic genes and the delivery of nanomaterial-loaded CXCL12 can promote neovascularization, thus providing new strategies for chronic wound healing.

Among the two clinical studies that used plerixafor, the one by Bonora et al. (2020) had a small sample size and significant wound heterogeneity in patients, and most of them had undergone vascular reconstruction surgery, so the drug effect may not have been noticeable. In addition, plerixafor has a short half-life, and the increase in HSPC levels caused by a single injection (0.24 mg/kg) cannot meet long-term needs. The study did not test the level of CXCL12 to assess whether its expression had been impaired before treatment. This study revealed that we must clarify the functional changes in related cells in the diabetic environment. The other study by Ahmadi et al. (2023) did not elucidate the molecular mechanisms by which MRG-001 exerts its immunomodulatory and tissue regenerative effects, and the optimal dosage and specific dosing regimen need to be refined. The findings of studies on CXCR4 agonists need to be validated in human subjects. UCUF-728 was observed briefly in animal experiments and must be mined for biomarkers that can predict its efficacy (Xu J. et al., 2022). The optimal dose and safety range of UCUF-965 must be determined by refining pharmacokinetic and pharmacodynamic studies and dose-effect experiments (Peddibhotla et al., 2023). Both substances may accelerate wound healing by directly promoting cell migration; however, this may increase the risk of fibrosis and scar formation. While experimental evidence from animal studies suggests that CXCR4 agonists may be safer and more efficacious alternatives to antagonists, the data are not robust and lack confirmation from clinical trials. Researchers must elucidate the mechanism of the dual action of AMD3100 and the pro-healing effects of UCUF-728 and UCUF-965 to determine the dose and safety ranges for the switch of action and the threshold for achieving scarless healing. A comprehensive evaluation of its effectiveness, identification of potential side effects, and long-term follow-up through multicenter, large-sample, randomized, double-masked, long-term clinical trials are also needed. The risk of systemic immune activation also needs to be assessed, even for topical applications, taking into account wounds at different stages of diabetes and of various degrees of severity. Thus, it can be combined with a responsive hydrogel on the basis of its ability to improve the stability and bioavailability of CXCR4 agents and monitor factors such as pH, ROS, and temperature to achieve controlled delivery and diagnostic integration. These findings may reveal broader prospects for clinical translation.

*Ex vivo* and *in vivo* studies have shown that variations in the wound microenvironment add complexity, highlighting the importance of customizing hydrogel dressings. When factors such as treatment duration, the frequency of dressing changes, and the likelihood of disease recurrence are considered, the overall cost of treatment may be lower than that associated with conventional dressings (Chen et al., 2023). Responsive hydrogels loaded with CXCR4 agents meet the clinical requirements for an ideal dressing. In the future, inspired by wound healing strategies such as NPWT, HBOT, EST, and phototherapy, a combination of pro-healing strategies could be achieved using responsive hydrogels loaded with CXCR4 agents as a bridge. Importantly, most hydrogels are mechanically poor and prone to rupture, and their mechanical properties need to be improved. Studies have shown the potential of injectable hydrogels to meet the need for filling irregular wounds (Ogle et al., 2018) and to resist damage to hydrogel dressings caused by external forces such as exercise. The diversity of raw materials and response factors has resulted in elevated standards for production, transport, storage, and batch production. Neither the market acceptance nor patient compliance of responsive hydrogels has been evaluated. Before initiating large-scale manufacturing, ethical review and authorization from the FDA and other pertinent agencies are mandatory. Strict purity and biocompatibility testing are required during production to ensure homogeneity. Before utilization, hydrogel dressings must undergo rigorous testing to ascertain the immunological tolerance of the raw materials. Moreover, continuous monitoring of adverse effects and systematic collection of patient feedback are essential to optimize dressing design.

In complex wound environments, the non-specificity of a hydrogel’s response may reduce its efficacy in promoting diabetic wound healing. The desired drug release rate for temperature-responsive hydrogels requires high sensitivity. In contrast, ROS-responsive hydrogels must be stable in non-ROS environments, and pH-responsive hydrogels must be soluble or degradable in wound-specific pH environments. For multiple-responsive hydrogels, each factor must function independently to avoid interference with the others, thereby facilitating the pursuit of synergistic effects and controlled drug loading. Consequently, rigorous preclinical investigation is paramount to meticulously calibrate the triggering conditions for each response mechanism, thereby establishing dependable clinical monitoring indicators and external control systems. These systems facilitate the precise evaluation of alterations in the wound microenvironment, ensuring personalized wound care and enhancing the efficacy of clinical translation. The prevalence of deleterious responses, including pain, allergic reactions, hemolysis and secondary infection, is minimal in animal experiments and clinical studies, irrespective of whether the hydrogel is ordinary or responsive. Nevertheless, regarding patient adherence, subsequent research must comprehensively consider potential side effects to ensure the safety of clinical translation. The present animal model cannot adequately replicate the intricacy of diabetic wounds, and the experimental period is considerably shorter than the actual development time of diabetic wounds. In future research, it is necessary to comprehensively integrate operability and research cost and select a model that mirrors the structure of human skin. It is also necessary to extend the administration and observation period of animal experiments, dynamically monitor the release pattern of the active ingredient of the CXCR4 agents in the experiments, and optimize the distribution ratio of each component of the responsive hydrogels through performance parameters to establish a reliable scoring system to assess the safety of long-term use. Importantly, prolonged usage of hydrogels can result in a gradual loss of functionality. The degradation products of these materials may be recognized as foreign substances by the immune system, thereby triggering an inflammatory response. Consequently, it is imperative to circumvent any potential toxicity by meticulously regulating the amount of monomer residue, judiciously incorporating cytotoxic additives, and prudently selecting stable cross-linking agents. The degree of cross-linking of GelMA hydrogels substantially influences the behavior of immune cells and fibroblasts during the wound healing process, with softer lo-GelMA promoting cellular infiltration and decreasing scar formation. Moreover, harder hi-GelMA increases the inflammatory response and degree of fibrosis (Butenko et al., 2024). This finding demonstrates that by adjusting the physical properties of the hydrogels, the cellular response can be directed, and this is a subject worthy of further exploration.

## 10 Conclusion

Delayed or nonhealing diabetic wounds severely affect patient quality of life and burden healthcare systems. The CXCR4/CXCL12 signaling pathway, crucial for mobilization, migration, and angiogenesis, is weakened in diabetic wounds. Various cellular and pharmacological approaches promote wound healing by enhancing stem cell mobilization, fibroblast and epithelial cell migration, immune regulation, and re-epithelialization. The CXCR4 antagonist AMD3100 aids wound healing by promoting mobilization but impairs healing by inhibiting migration and angiogenesis. This raises questions on its safety. In contrast, CXCR4 agonists like UCUF-728 and UCUF-965 are safer and show fewer physiological side effects. Responsive hydrogels, sensitive to pH, ROS, temperature, enzymes, light, or magnetism, have demonstrated safety and effectiveness in diabetic wound models. With interdisciplinary collaboration, these hydrogels, combined with CXCR4 agents and targeted wound therapies, offer a promising solution to the challenge of diabetic wound healing.
