# Correction: Mechanism underlying the involvement of CXCR4/CXCL12 in diabetic wound healing and prospects for responsive hydrogel-loaded CXCR4 formulations

**DOI:** 10.3389/fphar.2025.1646006

**Published:** 2025-07-01

**Authors:** Lingli Wang, Fengsong Nie, Zhaoyu Lu, Yang Chong

**Affiliations:** ^1^ Department of Clinical Nutrition, The Affiliated Hospital of Yangzhou University, Yangzhou University, Jiangsu, China; ^2^ Institute of Translational Medicine, Medical College, Yangzhou University, Yangzhou, Jiangsu, China; ^3^ Department of General Surgery, The Affiliated Hospital of Yangzhou University, Yangzhou University, Jiangsu, China

**Keywords:** diabetic wound, CXCR4, CXCL12, mobilization, migration, agonist, AMD3100, responsive hydrogels

**Affiliation 1**, Department of Clinical Nutrition, The Affiliated Hospital of Yangzhou University, Yangzhou University, Jiangsu, China, was omitted from the paper. This affiliation has now been added for authors **Lingli Wang** and **Yang Chong**. As such, Institute of Translational Medicine, Medical College, Yangzhou University, Yangzhou, Jiangsu, China, previously listed as affiliation 1 has been demoted to **Affiliation 2**. Furthermore, Department of General Surgery, The Affiliated Hospital of Yangzhou University, Yangzhou University, Jiangsu, China, previously listed as affiliation 2 has been demoted to **Affiliation 3**. All the affiliation numbers have been updated accordingly in the author list.

The authors apologize for these errors and state that this does not change the scientific conclusions of the article in any way. The original article has been updated.

